# Effect of oral alpha lipoic acid in treatment of OSMF

**DOI:** 10.6026/9732063002001401

**Published:** 2024-10-31

**Authors:** Shafeem Khan AK, Vikas Kulkarni, Abhay D. Havle

**Affiliations:** 1 Department of ENT, Krishna Institute of Medical Sciences, Karad - 415110, Maharashtra, India; 2 Department of Otorhinolaryngology, Krishna Institute of Medical Sciences, Karad - 415 539, Maharashtra, India

**Keywords:** Alpha lipoic acid, oral submucous fibrosis, thioacetamide, vernier caliper, hyaluronidase

## Abstract

Oral submucous fibrosis (OSMF) is a potentially cancerous disorder that affects the oral mucosa and causes a range of symptoms. Therefore it is of interest to assessing the function of oral alpha lipoic acid as a therapy for OSMF is intriguing. Hence, fifty patients with OSMF symptoms were evaluated both before and after treatment. They were split into two groups: group A, which served as the control, received 1 ml steroid injection of thioacetamide and 1500 IU of hyaluronidase (HYAL) weekly along with the oral antioxidant α-lipoic acid for three months. Group B, which served as the case study, also received 3 months of steroid injection of thioacetamide and HYAL. Patients in group B were recalled for a clinical examination using a vernier caliper, and visual analog scale (VAS) and fibrous bands (FB) were assessed at the 1^st^, 3^rd^ and 6^th^ month. We found that controls had a comparable distribution pattern across visits when compared to the case. Treatment with alpha lipoic acid in conjunction with intralesional injection of thioacetamide and HYAL significantly reduced the severity of symptoms, especially burning sensation and mouth opening, in the case group over time, as previously determined.

## Background:

Oral sub-mucous fibrosis (OSMF) is a clinical condition that is difficult to manage and is associated with a variety of devastating effects. The patient may experience severe physical and mental distress as the condition advances [1, 2]. There are a variety of treatment approaches that have been supported for the purpose of managing the symptoms as well as treating the aberrant fibrotic tissue that develops as the disease advances [3]. Still, no single treatment regime has shown to be completely acceptable, yet, since the pathophysiology of this disease is still not completely known [3, 4]. Usually stopping the practice of chewing areca/betel nut may effectively alleviate the symptoms, particularly if the issue is identified before to the onset of tissue fibrosis symptoms [3]. But once trismus is well-established, the severity of the condition's functional manifestation as well as its clinical appearance determines the course of treatment [1, 4]. Various treatments for OSMF include steroids and hyaluronidase. Steroids work by suppressing the immune system and have anti-inflammatory effects, whereas hyaluronidase breaks down hyaluronic acid, decreasing collagen production. The combination of injecting hyaluronidase with oral colchicine has displayed superior results in enhancing mouth opening and alleviating symptoms when compared to other treatments [5]. The addition of α-lipoic acid (AL-A) to intra-lesional steroids and hyaluronidase has demonstrated potential in the management of OSMF. Alpha lipoic acid supplementation has resulted in the improvement of burning mouth sensation (BMS) and mouth opening in patients with OSMF, indicating its potential as a helpful supplementary therapy. The combination of oral colchicine with intralesional IL-HYAL or thioacetamide, along with additional Alpha lipoic acid, has shown potential in alleviating symptoms and increasing mouth opening in OSMF patients. Therefore, it is of interest to report the effect of oral α- lipoic acid as a supplement to the intralesional injection (IL-I) of triamcinolone acetonide (TAA) and hyaluronidase (HYAL) in OSMF.

## Materials and Methods:

The current single-blind observational study was conducted in the E.N.T Department of Krishna Hospital (Deemed to be University), Karad starting from May 2022 - May 2023 with a total of 50 patients divided into 2 groups with 25 patients each. The baseline investigations such as the mouth opening, color, and burning sensation of the oral mucosa (OM) were assessed in groups A and B before and after the administration of treatments. The intralesional injection was administered in the soft palate and the fibrous bands (FB) formed anterior to the anterior pillars, at multiple sites bilaterally. Addition to above, group A was treated with a combination of a weekly 1 ml steroid injection (SI) of thioacetamide and 1500 IU of HYAL, administered intralesional injection using the multiple puncture method for 3 months. They were also given an oral antioxidant, alpha lipoic acid (600mg), once daily for 3 months. Whereas, group B received treatment solely with a combination of a weekly 1 ml SI of thioacetamide and HYAL that is administered intralesional injection using the multiple puncture method for 3 months.

This group did not receive the antioxidant alpha lipoic acid and served as the control group. The clinical examination included measuring the inter-incisal distance using a Vernier caliper, assessing the presence of fibrous bands, and recording the burning sensation reported by patients using a 10-point visual analog scale (VAS). Following a thorough clinical examination, each patient had an in-depth interview with specific reference to the kinds, frequency, and duration of oral abusive practices. The burning sensation reported by patients during the presenting visit as well as any subsequent follow-up appointments was documented. Before beginning the intervention, all patients were asked to stop their oral bad habits with follow up at 1^st^, 3^rd^ and 6^th^ month intervals.

## Inclusion criteria:

[1] Both genders.

[2] Above the age of 13 years.

[3] Those who were clinically diagnosed with fibrotic bands over or oral mucosa, decrease mouth opening, protrusion of tongue, BS, recurrent ulceration, dryness of mouth.

[4] Those biopsy confirmed OSMF.

## Exclusion criteria:

[1] Other pre-cancerous & cancerous lesion of oral cavity.

[2] Severe trismus.

[3] Inter incisor distance less than 10 mm.

[4] OM disorder.

[5] Suffering from TMJ problems.

[6] Prior history of mandibular fracture.

[7] Systemic Disorder.

[8] Unwilling to participate.

[9] Undergoing Chemo-radiotherapy.

## Statistical analysis:

SPSS version 27 was used to analyze the data. Chi-square test were used for patient demographics. Paired t-test was used to study the impact of treatment.

## Results:

Table 1 shows that, the highest percentage was seen in the 21-30 age group (32.00%), followed by the 31-40 age group (24.00%). Controls mirror a similar distribution, with 32.00% in the 21-30 age range and 24.00% in both the 31-40 and 41-50 age brackets. Table 2 shows that, the majority of both cases (72.00%) and controls (64.00%) were males, whereas females account for 28.00% of cases and 36.00% of controls. Table 3 shows that, the mild test category recorded 4 cases and 5 controls, the moderate category saw 10 cases and 13 controls, and the severe category had 11 cases and 7 controls. Table 4 shows that, in the 1^st^ month visit, there were 13 cases and 9 controls; in the 3^rd^ month visit, there were 10 cases and 8 controls; and in the 6^th^ month visit, there were 2 cases and 8 controls. Table 5 shows that, in the 1^st^ month, there were 9 cases and 9 controls; at the 3^rd^ month visit, there were 8 cases and 9 controls; and by the 6-month visit, there were 8 cases and 7 controls. Table 6 shows that, in the 1^st^ month visit, there were 3 mild cases, 12 moderate cases, and 10 severe cases. By the 3^rd^ month visit, these numbers shifted slightly with 4 mild cases, 15 moderate cases, and 6 severe cases. In the 6^th^ month visit, the distribution was 5 mild cases, 17 moderate cases, and 3 severe cases. Controls showed a similar distribution pattern across visits.

## Discussion:

In our study, age group of 13-20 years there were 3 cases (12.00%) and 3 controls (12.00%). For the 21-30 age groups, both cases and controls had 8 individuals each, accounting for 32.00% in both groups. Among those aged 31-40, there were 6 cases (24.00%) and 6 controls (24.00%). In the 41-50 age group, there were 5 cases (20.00%) and 6 controls (24.00%). Lastly, for individuals above 50 years, there were 3 cases (12.00%) and 2 controls (8.00%). The gender distribution among cases and controls is as follows: Among the cases, 18 were male (72.00%) and 7 were female (28.00%). In the control group, 16 were male (64.00%) and 9 were female (36.00%). In the study by Verma *et al.* it was noted that 40 cases (39.21%) were in the age group of 21-30 years, while the least affected age group was 51-60 years, comprising 8 cases (7.84%). A total of 204 patients were included in the study. Out of these 140 (68.62%) were males and 64 (31.38%) were females [6]. Extent of severity in our study showed among participants showed that both groups had a similar number of participants experiencing mild level severity, with 4 cases and 5 controls. However, differences emerged in the other severity levels. In the moderate level severity category, the control group had a higher number of participants (*i.e.*) 13 compared to the case group (*i.e.*) 10, indicating that moderate level severity was more common among the controls. Conversely, the case group had more participants experiencing severe level of severity, with 11 cases compared to 7 controls. This suggests that severe level of severity was more prevalent in the case group. Overall, while both groups had participants across all severity levels, the control group showed a higher prevalence of moderate severity. In contrast, the case group had a higher prevalence of severe level of severity. Moreover, the most significant change is observed by the 6^th^ month visit, where only 2 cases reported a burning sensation, compared to 8 in the control group. This suggests that the treatment had a substantial impact on reducing the burning sensation over time for the case group, while the control group did not experience a similar reduction.

The study done by Shrinivas *et al.* revealed that a large majority of participants experienced relief from symptoms, including restricted mouth opening, burning sensation and blanching of the mucosa (BOM). These findings underscore the effectiveness of the intervention in alleviating these common manifestations of the condition [7]. Nilesh *et al.* observed an increase in mouth opening in all patients at the 2nd-month follow-up after multidrug therapy. Group 1's mean interincisal distance increased from 36.6 mm to 38 mm, Group 2's from 28.30 mm to 32.23 mm, and Group 3's from 17.8 mm to 20.2 mm. The mean increases were 1.3 mm, 3.9 mm and 2.4 mm, respectively, all statistically significant. Additionally, there was a significant decrease in VAS scores for burning sensation, with reductions of 1.42, 2.46, and 1.86 in Groups 1, 2 and 3, respectively (p < 0.00001 for all) [8]. In their study, Veedu *et al.* found that the HYAL group had the highest number of severe cases based on clinical staging. However, this group demonstrated the most significant improvement, with a mean increase of 6.67 ± 3.74 mm. The combination group showed moderate improvement, averaging a mean increase of 5.8 ± 2.60 mm, while the dexamethasone group exhibited minimal IP, with a mean increase of 4.27 ± 1.58 mm [9]. Shrinivas *et al.* observed that in Group I before treatment, mouth opening was limited. Following treatment, there was an improvement in mouth opening. Similarly, Group II, Group III, and Group IV showed varying degrees of improvement in mouth opening post-treatment. Also, James *et al.* demonstrated that combining HYAL with dexamethasone resulted in a significant improvement in mouth opening (6 ± 2 mm), highlighting the effectiveness of this intervention [7]. In our study, for mild severity we found that, there was an increase in the number of participants over time in the case group, from 3 at the 1^st^ month visit to 5 by the 6^th^ month visit. The control group started with 4 participants on the 1^st^ month visit, increasing slightly to 5 participants by the 3^rd^ and 6^th^ month visits. For moderate severity, the case group showed a consistent increase in the number of participants, from 12 at the 1^st^ month visit to 17 by the 6^th^ month visit. The control group had a smaller increase, from 13 participants at the 1^st^ month visit to 14 participants at the 3^rd^ and 6^th^ month visits. For severe severity, there was a notable decrease in the case group, from 10 participants at the 1^st^ month visit to just 3 by the 6^th^ month visit. The control group showed a decrease from 8 participants at the 1^st^ month visit to 6 participants by the 3^rd^ and 6^th^ month visits. In a study, done by Rao *et al.* they observed that the changes in stage were observed from pre-treatment to post treatment in the alpha lipoic acid group. Group I showed improvements in Stage I and Stage II, with a statistically significant difference noted. Group II also exhibited changes in Stage I and Stage II, although the difference was not statistically significant. They concluded that the antioxidant, alpha lipoic acid, has a definitive protective role as demonstrated in this study, and it can certainly be recommended for clinical use [10]. Naik *et al.* concluded that the intralesional injection with placental extract and thioacetamide with HYAL are equally effective in treating trismus of OSMF [11].

## Conclusion:

The gender distribution indicated a higher prevalence of males in both cases (72.00%) and controls (64.00%). Treatment effectively reduced burning sensation among cases over time, from 13 participants at the 1^st^ mouth opening nth to 2 by the 6^th^ mouth opening nth, while the control group remained relatively stable. Both cases and controls showed modest improvement in mouth opening over the study period.

## Figures and Tables

**Table 1:** Age distribution

**Age group**	**Case**	**Percentage**	**Control**	**Percentage**
13-20	3	12.00%	3	12.00%
21-30	8	32.00%	8	32.00%
31-40	6	24.00%	6	24.00%
41-50	5	20.00%	6	24.00%
above 50	3	12.00%	2	8.00%
Total	25	100.00%	25	100.00%

**Table 2:** Gender distribution

	**Case**		**Control**	
**Gender**	**No of cases**	**Percentage**	**No of cases**	**Percentage**
Male	18	72.00%	16	64.00%
Female	7	28.00%	9	36.00%
Total	25	100.00%	25	100.00%

**Table 3:** Severity distribution

**Test**	**Cases**	**Control**
Mid	4	5
Moderate	10	13
severe	11	7
Total	25	25

**Table 4:** BS Distribution

**Burning Sensation**	**Cases**	**Control**
1^st^ month visit	13	9
3^rd^ month visit	10	8
6^th^ month visit	2	8
Total	25	25

**Table 5:** R-MO distribution

**Restricted mouth opening**	**No of cases**	**Control**
1^st^ month visit	9	9
3 month visit	8	9
6 month visit	8	7
Total	25	25

**Table 6:** Extent of severity

	case			**control**		
Test	1^st^ month visit	**3^rd^ month visit**	**6^th^ month visit**	**1^st^ month visit**	**3^rd^ month visit**	**6^th^ month visit**
mild	3	4	5	4	5	5
moderate	12	15	17	13	14	14
severe	10	6	3	8	6	6
Total	25	25	25	25	25	25

## References

[1] Arakeri G, Brennan PA (2013). British Journal of Oral and Maxillofacial Surgery..

[2] IARC Working Group on the Evaluation of Carcinogenic Risks to Humans. (2004). IARC monographs on the evaluation of carcinogenicrisks to humans..

[3] Kerr AR (2011). Oral diseases..

[4] Chole RH (2012). Oral oncology..

[5] Arakeri G (2017). Journal of Oral Pathology & Medicine..

[6] Verma PK (2022). Indian Journal of Otolaryngology and Head & Neck Surgery..

[7] Shrinivas (2018). Med Pulse-Int J Dent..

[8] Nilesh K (2021). Annals of Dental Specialty..

[9] Veedu RA (2015). Oral surgery, oral medicine, oral pathology and oral radiology..

[10] Rao PK. (2010). Journal of cancer research and therapeutics..

[11] Naik SM (2012). International Journal of Head and Neck Surgery..

